# DRD4 Genotype and the Developmental Link of Peer Social Preference with Conduct Problems and Prosocial Behavior Across Ages 9–12 Years

**DOI:** 10.1007/s10964-015-0289-x

**Published:** 2015-05-09

**Authors:** J. Marieke Buil, Hans M. Koot, Tjeert Olthof, Kelly A. Nelson, Pol A. C. van Lier

**Affiliations:** Department of Developmental Psychology, VU University Amsterdam, Van der Boechorststraat 1, 1081 BT Amsterdam, The Netherlands; EMGO Institute for Health and Care Research, Amsterdam, The Netherlands; Avera Institute for Human Genetics, 3720 W. 69th Street, Suite 200, Sioux Falls, SD USA

**Keywords:** Gene–environment interaction, DRD4, Peer social preference, Conduct problems, Prosocial behavior, Differential susceptibility

## Abstract

The peer environment is among the most important factors for children’s behavioral development. However, not all children are equally influenced by their peers, which is potentially due to their genetic make-up. The dopamine receptor D4 gene (DRD4) is a potential candidate gene that may influence children’s susceptibility to the peer environment. In the present study, we explored whether variations in the DRD4 gene moderated the association between children’s social standing in the peer group (i.e., social preference among classmates) with subsequent conduct problems and prosocial behavior among 405 (51 % females) elementary school children followed annually throughout early adolescence (ages 9–12 years). The behavioral development of children with and without the DRD4 7-repeat allele was compared. The results indicated that children who had higher positive social preference scores (i.e., who were more liked relative to disliked by their peers) showed less conduct problem development in subsequent years relative to children who had lower positive social preference scores. In contrast, children who had more negative preference scores (i.e., who were more disliked relative to liked among peers) showed more conduct problem development in subsequent years, relative to children who had less negative preference scores. However, these effects only occurred when children had a 7-repeat allele. For children who did not have a 7-repeat allele, the level of social preference was not associated with subsequent conduct problems. No evidence for gene–environment interaction effects for prosocial behavior was found. The implications for our understanding of conduct problem development and its prevention are discussed.

## Introduction

In school, children have to function in a classroom for a significant amount of time every day, across the better part of their childhood and later adolescent years. As in every social setting, children evaluate classmates and form opinion on who they do and do not like. As a consequence of this evaluation, some children will become highly preferred and liked among many of their peers. These highly preferred children have been found to develop high-quality friendships (Parker and Asher 1993), have positive relationships with teachers (Hughes et al. 2006), and generally show favorable developmental outcomes such as prosocial behavior (Bierman and Erath 2006). However, the dark side of the peer evaluation process is that some children become disliked and poorly preferred by their classmates, which is a robust predictor of maladjustment. For instance, these children are at risk of peer victimization and friendlessness (Van Lier and Koot 2010) and poor support or rejection by teachers (Leflot et al. 2011). As such, it may come as no surprise that children who are poorly preferred by their peers are at risk of developing behavioral problems (Ladd 2006; Van Lier and Koot 2010).

Thus, there is a vast body of research linking children’s social standing among peers, also known as “peer social preference” (Coie et al. 1982), to childhood adjustment and maladjustment. However, individual differences in the predictive links are striking. Recent findings have suggested that the genetic make-up of children may be of relevance in understanding why children are more or less affected by their social environment (for a meta-analysis, see Bakermans-Kranenburg and van IJzendoorn 2011). That is, several studies have indicated that the dopamine receptor D4 gene (DRD4) may render children susceptible to environmental influences “for better and for worse” (Bakermans-Kranenburg and van IJzendoorn 2011, p. 39). According to this viewpoint, carriers of the 7-repeat allele (DRD4-7r) may be disproportionally susceptible for developing negative behavioral outcomes in an adverse environment, but are also more likely to respond with positive behavioral outcomes when in a favorable environment (Belsky and Hartman 2014). In the present study, we aimed to investigate the possible moderating role of DRD4 in the prospective association between low and high levels of peer social preference and the development of conduct problems and prosocial behavior, among children attending elementary school who were followed annually from age 9 to 12 years.

### DRD4 7-Repeat Allele and Environmental Influences

According to the differential susceptibility hypothesis (Belsky 1997; Belsky and Hartman 2014), some genetic variants may render individuals more malleable to negative as well as positive environments with respect to subsequent development, while other individuals—depending on their genetic make-up—are altogether less influenced by their environment. In a nutshell, this viewpoint proposes that, in order to increase reproductive fitness it makes evolutionary sense that some children are more susceptible to their environment than others (Belsky 1997; Belsky and Hartman 2014). That is, parents may (subconsciously or consciously) aim to modify children’s behavior so that it matches the environmental requirements. If the future environment is predicted correctly, a beneficial behavior-environment match occurs that may support the offspring’s health and reproductive fitness. However, given that future environmental circumstances are uncertain, for some children a mismatch occurs, potentially resulting in adverse outcomes. Thus, if within a family some children are born with a genetic disposition that renders them highly susceptible to their environment and others have a genetic disposition that renders them less susceptible, the probability that for all offspring such a detrimental mismatch takes place decreases (example adapted from Belsky 1997).

A potential candidate gene that may further our understanding of individual differences in sensitivity to the environment is the dopamine receptor D4 gene, DRD4 (Bakermans-Kranenburg and van IJzendoorn 2011; Belsky and Hartman 2014). DRD4 regulates dopamine receptor activity in the brain, particularly in brain regions of the mesocorticolimbic dopamine pathway (Oak et al. 2000). The neurotransmitter dopamine plays a major role in reward, punishment, attention and motivation mechanisms related to social interaction and learning. Furthermore, dopamine may signal the salience of social events and is a key factor in the imprinting of motivational importance to environmental factors (Trainor 2011).

The coding DNA sequence of DRD4 is highly polymorphic, resulting in receptor variants that may be functionally different. In this regard, the 48-bp tandem repeat (48-bp VNTR) in the third exon, consisting of 2–11 repeats, has received much research attention in behavior genetics. It has been shown that DRD4 has higher potency for dopamine-mediated coupling to adenylyl cyclase in the presence of the short 2-repeat and 4-repeat alleles, than when receptors are encoded by the 7-repeat allele, known as DRD4-7r (Oak et al. 2000; Schoots and Van Tol 2003). Decreased postsynaptic inhibition due to the 7-repeat allele results in lower dopaminergic tone and a suboptimal response to dopamine. This is associated with heightened reward-related reactivity in the ventral striatum and reward-related behaviors like impulsivity (Forbes et al. 2009). In addition, the mesocorticolimbic dopamine pathway is associated with the functioning of the anterior cingulate cortex, which is related to processing punishment and reward stimuli. Changes in dopamine levels due to the DRD4 polymorphism could thus enhance social-environmental signals related to reward and punishment (Posner and Rothbart 2009). Indeed, subjects with the 7-repeat allele show increased reactivity to social-environmental stimuli compared to subjects without this allele, as evidenced by findings from brain imaging, observational and experimental studies in humans and animals (Grady et al. 2013; Sheese et al. 2007). When confronted with emotional stimuli, carriers of the DRD4-7r allele were found to show more brain activity than non-carriers in brain regions associated with attention to and appraisal of negative emotional stimuli, as well as in brain regions involved in preparation for action (Gehricke et al. 2015). To the best of our knowledge, as of yet no studies have used functional brain imaging to investigate whether brain regions that are involved in reactivity and attention with regard to negative stimuli also apply to positive stimuli. However, observational research has indicated that individuals with the 7-repeat allele show heightened sensitivity to positive parenting environments when compared to individuals without this allele (Bakermans-Kranenburg and van IJzendoorn 2011). Together, these findings may suggest that individuals with a 7-repeat allele of the DRD4 gene are more susceptible to their environment than individuals without this allele, irrespective of whether this environment is positive or negative. Furthermore, some authors suggested that the dopaminergic system is key to the development of social behavior (Insel 2003). This statement is supported by the fact that on a behavioral level DRD4-7r has been related to aggression in children (Schmidt et al. 2002), to conduct problems and oppositional behavior in individuals with Attention Deficit Hyperactivity Disorder (ADHD; Holmes et al. 2002; Kirley et al. 2004), and to diminished levels of prosocial behavior (Anacker et al. 2013; DiLalla et al. 2009; Jiang et al. 2013).

A recent meta-analysis showed that children with less efficiently functioning dopamine-related genetic variants (of which DRD4 was the most studied gene) do worse in negative parental rearing environments than children without such alleles (Bakermans-Kranenburg and van IJzendoorn 2011). At the same time, the authors concluded that children with susceptibility alleles are also likely to profit most from positive rearing environments (Bakermans-Kranenburg and van IJzendoorn 2011). Despite that the results presented in that meta-analysis generally supported the differential susceptibility hypothesis, the study of differential susceptibility of DRD4 to the social environment is far from complete.

First, although gene–environment interaction (G × E) studies of DRD4 in the parenting context are fairly common, only a few studies focused on the peer environment (i.e., DiLalla et al., 2009; Kretschmer et al. 2013). As said, children in elementary school function in the presence of their peers for a large proportion of their day. Consequently, the peer environment becomes increasingly important for the development of school-aged children (Sroufe et al. 2009). None of the studies that investigated the peer environment × DRD4 interaction effects focused on the elementary school period. DiLalla et al. (2009) found that preschoolers carrying the DRD4-7r allele showed more aggression during peer-play in an environment where there was little peer aggression, while in a highly aggressive environment all children showed aggressive behavior regardless of genotype. No evidence of G × E was found for the association between peers’ prosocial behavior and children’s own prosocial behavior in that study. Kretschmer et al. (2013) focused on victimization and social well-being during adolescence as predictors of delinquency. These authors found that, in contrast to previous findings and their own hypotheses, the adolescents who did *not* have the DRD4-7r allele, as opposed to those who did have this allele, were more susceptible to the effects of victimization and social well-being. Thus, information on the elementary school peer environment is lacking and the scarce studies with regard to moderation by DRD4 genotype in the relation between peer experiences and (mal)adjustment have produced inconclusive findings.

Second, many previous studies have studied environmental variables that not all children will be exposed to on a daily basis and for the better part of the week, such as bully-victimization, intrusive parenting, or peer aggression (e.g., DiLalla et al. 2009; Kretschmer et al. 2013; Propper et al. 2007). It is currently not known whether moderating effects of DRD4 also extend to peer experiences that children will encounter on each typical school day. In the present study we therefore focused on children’s social preference among peers as the environmental factor of interest. Peer social preference in the classroom refers to the extent to which children are liked relative to disliked by their classmates. It is the result of a natural evaluation process that occurs in every social setting, for every individual within that setting (Coie et al. 1982; Rubin et al. 2006). Establishing a positive social standing in the larger peer-group is a key developmental task for children in elementary school, which facilitates a healthy behavioral development (Sroufe et al. 2009). Indeed, the impact of low social preference within the peer group on behavioral misconduct in children has been well documented (for overviews, see Parker et al. 2006; Rubin et al. 2006). However, and in accordance with the “for better and for worse” hypothesis, the influence of peer relations is multidirectional: being mostly disliked among peers may elevate the risk for the development of conduct problems and may hinder prosocial development; in contrast, being mostly liked may buffer against the development of conduct problems and may promote prosocial behavioral development (Ladd 2006; Twenge et al. 2007; Wentzel 2014; Wentzel and McNamara 1999; Witvliet et al. 2009). Therefore, by focusing on social preference as the environmental peer-factor of interest we aim to expand previous results found in the field of gene × peer environment interactions.

Third, and related to the previous argument, none of the previous studies focused on both negative and positive environments with regard to both negative and positive outcomes. The study by Kretschmer et al. (2013) focused on negative and positive peer environmental factors with respect to predicting negative behavioral outcomes. The study by DiLalla et al. (2009) focused on a positive peer environment with respect to predicting positive behavioral outcomes and a negative environment with respect to predicting negative behavioral outcomes. Other studies also focused on either the positive environment or the negative environment and/or either positive outcomes or negative outcomes (e.g., see examples in the overview of Bakermans-Kranenburg and van IJzendoorn 2011). However, less negative behavioral outcomes or even the absence of negative behavioral outcomes does not necessarily mean that behavioral outcomes are positive. This also applies vice versa: less positive behavioral outcomes or the absence of positive outcomes does not necessarily mean that behavioral outcomes are negative. The same holds for the environment: the absence of a negative environment or a less negative environment does not necessarily mean that the environment is positive, and vice versa. Ideally, the study of differential susceptibility includes both negative and positive environments as well as both negative and positive behavioral outcomes to test for all possibilities: (a) a negative environment predicting more positive behavioral outcomes and less negative behavioral outcomes and (b) a positive environment predicting less positive behavioral outcomes and more negative behavioral outcomes. To this end, we focused on peer social preference as our environmental factor of interest and conduct problems and prosocial behavior as our behavioral outcomes of interest. Peer social preference encompasses both a risk (i.e., negative social preference scores: children who are more disliked relative to liked) and a protective end (i.e., positive social preference scores: children who are more liked relative to disliked). Thus, this allows for a comprehensive test of the differential susceptibility hypothesis. That is, moderation by DRD4 genotype in both the “for better” and the “for worse” direction can be tested by including both positive and negative peer environmental factors with respect to predicting both positive and negative outcomes.

Lastly, many previous studies suffered from design limitations because most were cross-sectional or longitudinal prediction studies that were built upon the assumption that children’s environment predicts subsequent behavior and not vice versa. However, previous studies have shown that associations between social preference and behavior may be bidirectional: children’s social standing among peers may influence their behavior and their behavior may influence their social preference among peers (e.g., Van Lier and Koot 2010). Thus, when developmental models do not account for the possibility of these bidirectional effects, the direction of influence between environmental and behavioral factors may be obscured. Furthermore, by using the participants as their own controls, our longitudinal study in which the behavioral and environmental factors are assessed in parallel over 4 years enables investigating whether behavior has changed from a prior baseline level after experiencing low or high social preference.

## Present Study and Hypotheses

Using a sample of mainstream elementary school children (*N* = 405) in which social preference, prosocial behavior, and conduct problems were assessed in parallel, annually across ages 9–12 years (four waves), we aimed to extend previous research on the moderating role of DRD4 in four ways. First, we focused on the peer environment in elementary school children, thereby extending studies on parental environmental factors as well as studies focused on the peer environment in kindergarten and adolescence. Second, we focused on a peer environmental factor that all children experience on a daily basis for the better part of the week, namely peer social preference. We thereby expand previous research that used peer factors that likely not all children are exposed to. Third, by focusing on both negative and positive peer environmental factors in predicting both negative and positive behavioral outcomes, we tested the differential susceptibility hypothesis in a comprehensive manner. Lastly, we investigated potential G × E effects in a longitudinal design where children were followed over 4 years, which enabled us to investigate the direction of influence between the behavioral and environmental constructs.

We started by investigating whether positive social preference scores and negative social preference scores would be prospectively associated with conduct problems and prosocial behavioral development, above and beyond possible direct effects of DRD4 on the environmental and behavioral variables, as well as above and beyond potential opposite effects (i.e., behavior affecting social preference). We hypothesized that children who had higher positive preference scores would have lower levels of conduct problems and higher levels of prosocial behavior in subsequent years, relative to children with lower levels of positive preference scores. Furthermore, we expected these effects to be mirrored for children who had negative social preference scores. That is, we hypothesized that children who had more negative preference scores would have higher levels of conduct problems and lower levels of prosocial behavior in subsequent years, relative to children with less negative preference scores (hypothesis 1). Within these models, direct associations between DRD4 and social preference scores as well as between DRD4 and behavioral outcomes were explored.

Next we examined our main hypothesis, namely whether the prospective association between peer social preference and behavioral development varied as a function of DRD4 polymorphisms. In line with the differential susceptibility hypothesis, we tested whether the potential moderation by DRD4 occurred “for better and for worse” (hypothesis 2). Specifically, we hypothesized that children who had higher positive preference scores would have lower levels of conduct problems and higher levels of prosocial behavior in subsequent years, but in both cases particularly when they had a DRD4-7r allele (i.e., G × E “for better”). In addition, we expected that particularly for children with a DRD4-7r allele more negative preference scores would be related to subsequent higher levels of conduct problems and lower levels of prosocial behavior (i.e., G × E “for worse”).

## Method

### Participants

Participants were children attending 48 different mainstream elementary schools and were part of two longitudinal research projects on children’s social, emotional and behavioral development in the Netherlands. These research projects were conducted by the department of Developmental Psychology, VU University Amsterdam. Parental consent for participation was obtained for a total of 1091 children. In the first project, schools were recruited from two urban areas in the western part of the Netherlands and one rural area in the eastern part of the Netherlands. A convenience sample was utilized in which the first 30 schools that accepted our invitation to participate in the project were included. In the other project, eighteen schools from the northern and the eastern part of the Netherlands were recruited via municipal health services. In both projects, all children were followed annually across elementary school. Additional information on the participants, design, and procedures is provided elsewhere (Gooren et al. 2011; Menting et al. 2011). The ethic review boards of the Erasmus University Rotterdam and the VU University Amsterdam approved the projects. In first and second grade, a preventive intervention targeting problem behavior (either the Good Behavior Game; Barrish et al. 1969; or PATHS curriculum; Kusché and Greenberg 1994) was implemented in which approximately 60 % of the children participated, with the remaining 40 % serving as controls. To prevent confounding by intervention effects, data covering ages 9–12 years (grades 3–6, four waves) were used in the present study. Moreover, all estimates were controlled for potential long-term intervention effects and three-way interactions including condition (intervention or control; G × E × condition) were tested. More detailed information about both interventions can be found in “Appendix 1”.

At age 13, children were asked to provide DNA through a saliva sample. Children and parents who granted permission were eligible for inclusion in the present study (*N* = 406; 51 % girls). DRD4 genotyping was successful for 405 out of the 406 subjects. Of these, 143 (35 %) subjects carried one or two 7-repeat alleles (referred to as DRD4-7r) and 262 (65 %) subjects carried no 7-repeat alleles (referred to as DRD4-no7). Of the DRD4-no7 group, all but 2 children carried either a 2-repeat allele or a 4-repeat allele. More details on the distribution of the DRD4 polymorphisms and the assignment to groups is provided in “Appendix 3”.

Eighteen percent of the children came from low socioeconomic status (SES) families. Furthermore, 87 % of the present sample had a Dutch/Caucasian background, 3.8 % were Moroccan, 3.8 % were Surinamese, 2 % were from the Netherlands Antilles, and 3.4 % of the children came from other ethnical backgrounds (i.e., Turkey, Somalia, Pakistan, Iraq, Congo-Kinshasa, and Sri Lanka). Given that the DRD4-environment interaction may be dependent on race (e.g., Propper et al. 2007), we examined whether results changed when only native Dutch (i.e., Caucasian) children remained in the sample. In addition, because the developmental relation between peer experiences and subsequent behavioral development may differ for boys and girls (Moffitt et al. 2001; Van Lier and Koot 2010; Witvliet et al. 2009) and that moderating effects of DRD4 may be influenced by the child’s sex (Froehlich et al. 2007), we investigated potential sex differences in the moderation by DRD4 (i.e., G × E × sex).

Participants who declined participation in DNA collection did not differ from those who conceded with participation on average levels of conduct problems, (*F*(1, 973) = 2.49, *p* = .12) or negative social preference scores (*F*(1, 1089) = 1.48, *p* = .22) over ages 9–12 years. However, children who declined participation compared to children who participated had slightly lower average levels of prosocial behavior (*F*(1, 972) = 11.44, *p* < .01, *η*^2^ = .01; *M* = 2.87, *SD* = 0.57 for children who participated, *M* = 2.74, *SD* = 0.62 for children who declined participation), as well as slightly lower levels of positive social preference scores (*F*(1, 1010) = 6.27, *p* < .05, *η*^2^ < .01; *M* = 0.23, *SD* = 0.16 for children who participated, *M* = 0.20, *SD* = 0.17 for children who declined participation) over ages 9 to 12 years. During the follow-up period used in the present study, data of 91 % of the children were complete for at least two measurement moments. Missing data was due to retention, moving to another school, or because of absence during the measurements. Children with missing data did not differ from children with complete data on any of the study variables in third grade, indicating that there was no evidence for selective attrition during the period investigated in the present study.

### Measures

#### Teacher Ratings of Conduct Problems

Teacher ratings of conduct problems were assessed annually with the conduct problems scale from the Problem Behavior at School Interview (PBSI; Erasmus 2000). The PBSI is a face-to-face interview in which teachers rated pupils’ behavior on a five-point Likert-scale ranging from 0 (never applicable) to 4 (often applicable). Conduct problems were assessed by 12 items (range α over the assessments = .90–.92). Sample items include: “attacks other children physically”, “bullies”, “steals”, “destroys property belonging to other children”, “is absent from school without permission”, “curses or swears”. Item scores were averaged, resulting in a scale ranging from 0 to 4.

#### Teacher Ratings of Prosocial Behavior

Teacher ratings of prosocial behavior were assessed annually with the prosocial behavior scale from the Social Experiences Questionnaire (SEQ-T; Crick and Grotpeter 1996). During a face-to-face interview teachers rated pupils’ behavior on a 5-point Likert scale ranging from 0 (never applicable) to 4 (often applicable). Prosocial behavior was assessed by 4 items (range α over the assessments = .75–.83). Sample items include: “Comforts a child who is sad” and “Is nice to other children”. Item scores were averaged, resulting in a scale ranging from 0 to 4.

#### Peer Nominations on Social Preference

Peer nominations on social preference were obtained by asking children to nominate an unlimited number of children in their classroom whom they liked most and whom they liked least. The “liked least” scores of each child were subtracted from his or her “liked most” scores to obtain a social preference score. This score was divided by the total number of children in the classroom, minus one (it was not allowed to nominate oneself), resulting in a score ranging from −1 (disliked by all classmates and liked by none) to +1 (liked by all classmates and disliked by none). This procedure was adapted from the protocol described by Coie et al. (1982). Social preference is generally regarded as a reliable and valid measure of sociometric status (Rubin et al. 2006). We then differentiated between children with *positive social preference scores*, that is children who were more liked relative to disliked and children with *negative social preference scores*, that is children who were more disliked relative to liked. Negative social preference scores were then multiplied by −1 such that higher scores reflected a more negative social preference score. Children who were equally liked as disliked or who were not nominated at all (between 3.3 and 6.5 % of all children throughout ages 9–12 years) received a score of zero.

### Covariates

#### Children’s Sex

Children’s sex was dummy coded as 0 = female, 1 = male.

#### Household Socioeconomic Status (SES)

SES was measured through parental occupation in third grade. Father’s and mother’s occupations were classified into one of five levels (0 = unemployed, 1 = elementary level, 2 = lower level, 3 = medium level, 4 = higher level). Levels of occupation were assigned according to the Dutch Working Population Classifications of Occupations Scheme (Statistics Netherlands 2001), which is based upon the International Standard Classification of Occupations (ISCO; International Labour Organization 1987a, b). The highest occupation level (from father or mother) was considered to reflect household SES. Household SES was then dummy coded as 0 = medium to higher level SES, 1 = unemployed to lower level SES.

#### Intervention Status

Intervention status was dummy coded as 0 = no intervention, 1 = intervention.

#### Genotyping of VNTR in Exon 3 of DRD4

DNA was extracted from saliva using the Oragane™ DNA Self-collection Kit according to the manufacturer’s instructions (DNAGenotek, Ottawa, Ontario, CAN). The 48 base pair VNTR in exon 3 of DRD4 (2–11 repeats) was genotyped using PCR and fragment analysis on a 3130 Genetic Analyzer (Life Technologies, Carlsbad, CA). The PCR assay was a modification of the method by Boór and colleagues (Boór et al. 2002). In accordance with previous studies (e.g., Kretschmer et al. 2013), children were coded as DRD4-7r (at least one allele had 7-repeats) or DRD4-no7 (no 7-repeat alleles).

### Statistical Approach

**Fig. 1:**
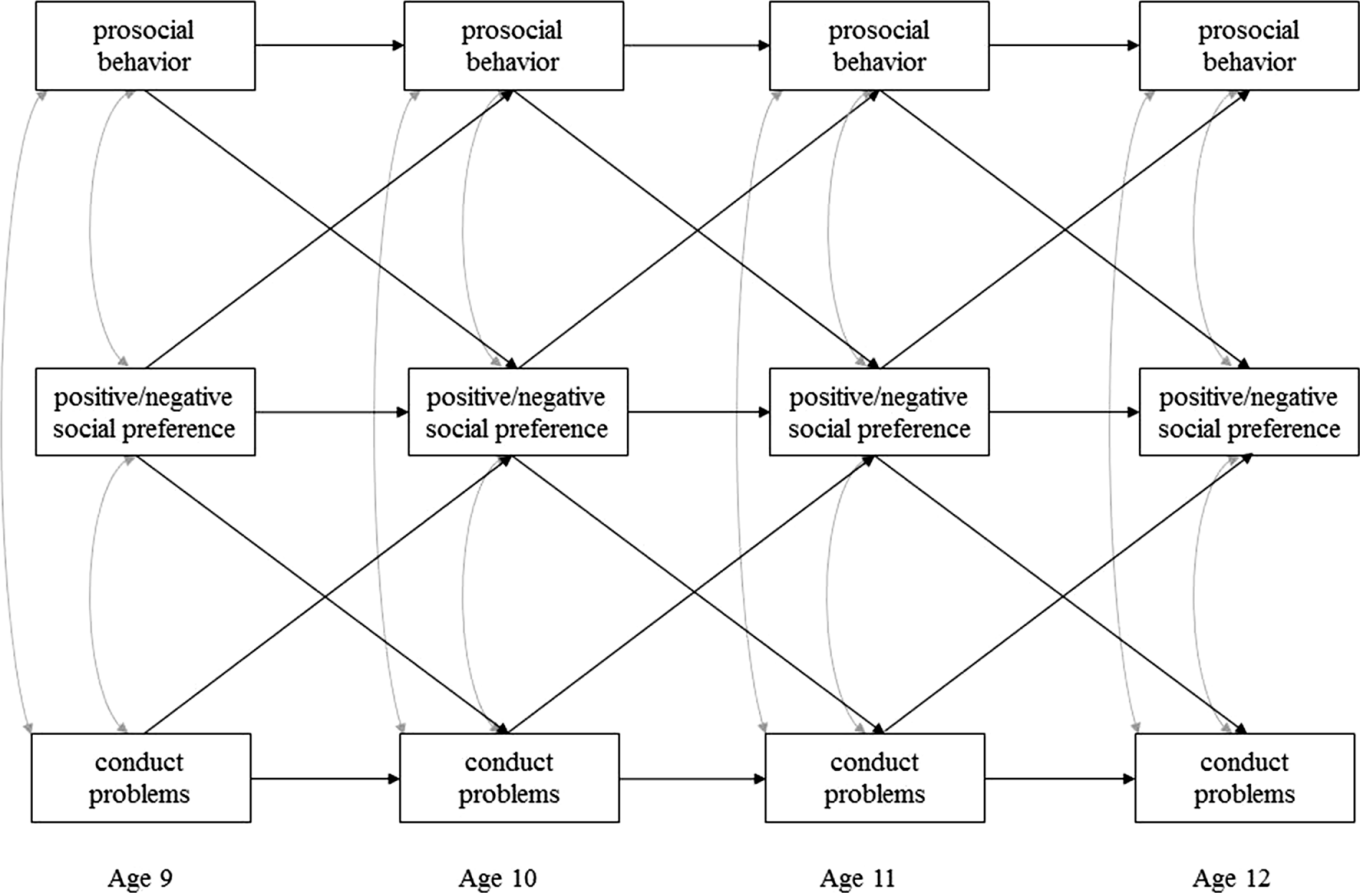
Illustration of the model used for hypotheses testing. This model was tested for positive social preference and negative social preference separately

Autoregressive cross-lagged models (Jöreskog 1970) were used to test our two hypotheses. Models were fitted in Mplus 6.11, Los Angeles, California (Muthén and Muthén 1998–2011). We aimed to test links between social preference scores, conduct problems and prosocial behavior in two separate models. That is, we specified one model for links between positive social preference scores and behavioral development and another model for links between negative social preference scores and behavioral development. Within each model, autoregressive paths from ages 9 to 12 years tested for stability within the environmental and behavioral constructs, while cross-lagged paths assessed the developmental links between these constructs (see Fig. 1 for an illustration). All estimates were controlled for potential long-term intervention effects, SES status and sex.

#### Power Analysis

Given that statistical power is a major concern in modern behavioral genetics (Duncan and Keller 2011), we conducted an a priori Monte Carlo simulation study to ensure that power was sufficient given our models and sample size before starting with testing our hypotheses. Statistical power is the probability of detecting a significant result given that the alternative hypothesis (in our study: that particularly children with a 7-repeat allele are susceptible to the peer environment “for better and for worse”) is true. Low statistical power is problematic, because it implies that true findings are likely to be missed (type II error) and because low power increases the proportion of significant results that are published, but that are actually false (type I error).

#### Monte Carlo Simulation

In an a priori Monte Carlo analysis, data are generated from a population with hypothesized parameter values. Then, a large number of samples are drawn and a model is estimated for each sample. Parameter values and standard errors are averaged over the samples (Muthén and Muthén 2002). We expected effects for negative social preference and positive social preference to be similar, thus we only investigated power for the model including positive social preference. We used 10,000 replications to ensure that stability would be reached. Data for a multiple-group model were generated using the following population values (see also “Appendix 2”). For the DRD4-7r group as well as for the DRD4-no7 group, means and variances of variables were standardized to 0 and 1 respectively; the standardized regression coefficients for autoregressive paths of social preference, conduct problems and prosocial behavior were all 0.60; standardized regression coefficients of lagged paths from behavioral outcomes to social preference were 0.05 and −0.05 for prosocial behavior and conduct problems respectively; and standardized residual correlations were 0.10 between social preference and prosocial behavior and −0.10 for social preference and conduct problems and for conduct problems and prosocial behavior. For the DRD4-no7 group the standardized regression coefficients of the lagged paths from social preference to prosocial behavior as well as to conduct problems were 0. These values were chosen based upon Keith and colleagues’ consideration that within the social sciences estimates (i.e., standardized regression coefficients) <0.05 are too small to interpret, estimates ≥0.05 are small but meaningful, estimates ≥0.10 are moderate, and estimates ≥0.25 are large (Keith 2006; Keith and Cool 1992).

The focus of the power investigation in the multiple-group autoregressive cross-lagged model was the standardized regression coefficient of the lagged paths from social preference to prosocial behavior and to conduct problems for the DRD4-7r group. Different standardized regression coefficients were estimated, starting from 0.05 (which is a small, but meaningful effect; Keith 2006) until a power of 0.80 by *p* < .05 was reached. Results are in “Appendix 2”. These indicated that a power of 0.80 (*p* < . 05) would be reached when the standardized regression coefficients would be 0.12 for the link between positive social preference and subsequent prosocial behavior and −0.12 for the link between positive social preference and subsequent conduct problems. A beta of 0.12 indicates a moderate effect in the social sciences (Keith 2006), which we deemed both reasonable and relevant. Under the condition of no effect (i.e., *β* = 0) for the DRD4-no7 group, this results in a significant difference in slopes at *p* < .01 when standard errors are 0.10 for the DRD4-7r group and 0.01 for the DRD4-no7 group (which is a rather larger difference in SEs and thus a stringent test of differences between slopes). Furthermore, coverage for the parameters of interest was 0.94, which indicates that the 95 % confidence intervals of 94 % of the 10,000 replications include the population value of 0.12 (prosocial behavior) and −0.12 (conduct problems; see Table 4 in “Appendix 2”). Hence, we assumed power to be sufficient to test our hypothesis on G × E effects.

#### Hypotheses Testing

After sufficient power was assured, our two hypotheses were tested as follows. We first tested for the prospective influence of social preference on subsequent behavioral development. To this end, we started with a model that included autoregressive paths and cross-lagged paths, in addition to cross-sectional correlations between social preference and the behavioral phenotypes (models 1; see example in Fig. 1). We also included direct effects of genotype on the environmental and behavioral variables. This model allowed us to test bidirectional effects (i.e., whether positive/negative social preference scores added to behavioral development above and beyond possible prospective associations between behavioral development and subsequent environmental changes), cross-sectional correlations, and direct effects of DRD4 (hypothesis 1). We tested these models separately for positive social preference scores and negative social preference scores, but the development of prosocial behavior and conduct problems was estimated simultaneously. We then continued by testing whether recurring autoregressive and cross-lagged paths could be constrained to be equal over time in order to create parsimonious models (models 2).

Next, we tested our second and main hypothesis, namely whether DRD4 moderated the prospective link between social preference and behavioral development. The following hierarchy of nested model comparisons was applied to test for potential differences between DRD4-7r and the DRD4-no7 groups. Multiple-group models were used in which children with the DRD4-7r allele were compared to children with DRD4-no7 alleles. First of all, all parameters were freely estimated between the groups (models 3); next, we tested whether pathways that were not part of our hypotheses (i.e., autoregressive paths and paths from the behavioral constructs to the environment) were equal between groups (models 4); and lastly, we investigated our hypothesized G × E effects by testing whether paths between social preference and prosocial behavior (models 5) and between social preference and conduct problems (models 6) were equal between groups. As said, two models were tested: one for positive social preference scores and one for negative social preference scores. In order to support our second hypothesis, constraining autoregressive paths and paths from the behavioral constructs to social preference to be equal between DRD4-7r carriers and DRD4-no7 carriers (models 4) should not significantly decrease model fit, while constraining the pathways between social preference and behavioral phenotypes to be equal for DRD4-7r carriers and DRD4-no7 carriers (models 5 and 6) should result in a significant drop in fit. In each model testing step, constraints that did not result in a significant drop in model fit were remained in subsequent models.

Full Information Maximum likelihood estimation with robust standard errors (FIML, MLR-estimator) was used to account for missing data. We accounted for clustering of data within schools by using a sandwich estimator (Williams 2000). The Satorra–Bentler Chi square difference test was used to compare nested models (Satorra, 2000). Model fit was determined via the Comparative Fit Index (CFI; with values >.95 indicating acceptable fit), and the Standardized Root Mean Squared Residual (SRMR; with values ≤.08 being acceptable) (Hu and Bentler 1998; Marsh et al. 2004). We tested for potential sex-differences and differences due to intervention status in the moderation by DRD4 using three-way interactions (G × E × sex and G × E × condition, respectively). Furthermore, using the equation provided by Duncan and Keller (2011) we calculated the False Discovery Rate (FDR) from Monte Carlo power analyses. The FDR indicates the proportion of false discoveries (i.e., the proportion of false support for our hypotheses when this support actually represents type I errors).

## Results

### Descriptive Statistics

Distribution of the DRD4 polymorphisms was comparable to reported global repeat frequencies (see “Appendix 3”; Chang et al. 1996). Allele frequencies of DRD4 polymorphisms were analyzed from Hardy–Weinberg equilibrium (HWE) using *χ*^2^ tests. No deviations from HWE were detected, *χ*^2^ (2) = 0.20, *p* = .90.

Table 1 gives the means and *SDs* for study variables for boys and girls. Repeated measures analyses of variance (ANOVAs) indicated that from ages 9 to 12 years, boys had on average higher levels of conduct problems (*F*(1, 394) = 57.83, *p* < .001, *η*^2^ = .13), and lower levels of prosocial behavior (*F*(1, 395) = 80.50, *p* < .001, *η*^2^ = .17), than girls. In addition, boys had slightly lower levels of positive social preference scores (*F*(1, 362) = 15.30, *p* < .001, *η*^2^ = .04), and slightly higher levels of negative social preference scores (*F*(1, 363) = 10.69, *p* < .01, *η*^2^ = .03), than girls. Correlations between study variables in Table 1 indicated significant cross-time correlations of conduct problems, prosocial behavior, positive and negative social preference in the expected directions. Furthermore, repeated measures ANOVAs indicated that DRD4-7r and DRD4-no7 carriers did not differ in their average levels of conduct problems, prosocial behavior or social preference throughout ages 9 to 12 years.

**Table 1:** Correlations, means, and standard deviations for peer social preference and behavioral phenotypes

	1	2	3	4	5	6	7	8	9	10	11	12	13	14	15	16
*Positive social preference*																
1. Age 9	–															
2. Age 10	.70**	–														
3. Age 11	.49**	.65**	–													
4. Age 12	.35**	.48**	.63**	–												
*Negative social preference*																
5. Age 9	−.45**	−.34**	−.27**	−.29**	–											
6. Age 10	−.30**	−.43**	−.32**	−.30**	.53**	–										
7. Age 11	−.26**	−.35**	−.45**	−.41**	.53**	.59**	–									
8. Age 12	−.18**	−.24**	−.35**	−.47**	.49**	.47**	.69**	–								
*Conduct problems*																
9. Age 9	−.40**	−.37**	−.30**	−.25**	.48**	.43**	.29**	.34**	–							
10. Age 10	−.28**	−.37**	−.34**	−.26**	.43**	.45**	.37**	.36**	.66**	–						
11. Age 11	−.23**	−.24**	−.23**	−.10	.31**	.22**	.28**	.25**	.55**	.58**	–					
12. Age 12	−.18**	−.23**	−.28**	−.24**	.34**	.32**	.28**	.36**	.57**	.55**	.65**	–				
*Prosocial behavior*																
13. Age 9	.37**	.34**	.34**	.31**	−.33**	−.17*	−.19**	−.24**	−.53**	−.32**	−.35**	−.37**	–			
14. Age 10	.25*	.29**	.34**	.25*	−.38**	−.35**	−.25**	−.08	−.34	−.65**	−.32**	−.38**	.39**	–		
15. Age 11	.17**	.29**	.34**	.24**	−.17**	−.20**	−.23**	−.24**	−.29**	−.35**	−.44**	−.30**	.37**	.60**	–	
16. Age 12	.29**	.39**	.32**	.30**	−.26**	−.29**	−.24**	−.30**	−.29**	−.33**	−.29**	−.43**	.40**	.46**	.48**	
*Mean boys*	0.20	0.19	0.20	0.28	0.07	0.08	0.07	0.06	0.69	0.71	0.57	0.58	2.63	2.73	2.61	2.66
*SD boys*	0.19	0.19	0.20	0.22	0.13	0.16	0.15	0.14	0.62	0.69	0.58	0.60	0.66	0.69	0.71	0.73
*Mean girls*	0.27	0.26	0.28	0.34	0.03	0.03	0.03	0.03	0.36	0.30	0.24	0.25	3.00	3.09	3.16	3.15
*SD girls*	0.24	0.23	0.21	0.24	0.10	0.11	0.10	0.09	0.50	0.38	0.35	0.37	0.69	0.53	0.60	0.61

* Significant at *p* < .05; ** significant at *p* < .01; *** significant at *p* < .001

### Hypothesis 1: Social Preference and Behavioral Development

We started by investigating the prospective associations between peer social preference and behavioral outcomes over time. No moderation by DRD4 genotype was tested at this stage. Links between positive social preference scores, conduct problems and prosocial behavior and negative social preference scores, conduct problems and prosocial behavior were tested in two separate models (see Fig. 1). We fitted bivariate cross-lagged autoregressive models with stability paths and directional paths from social preference to behavior and vice versa, in addition to cross-sectional correlations. Direct effects between DRD4 and social preferences and between DRD4 and behavioral outcomes were also included in the models.

Results of model fitting are presented in Table 2. The two models fitted the data adequately according to fit indices (models 1; CFIs ≥ .95, SRMRs ≤ .06). Constraining recurring autoregressive and lagged paths to be equal over time (model 2) did not result in worsened model fit for any of the two models (see Table 2). Therefore, these time-constraints were retained in the models. Estimates for models 2 are displayed in Table 3. For conduct problem development, neither positive social preference scores nor negative social preference scores were related to subsequent conduct problem development, although trends were observed (i.e., *p* ≤ .08). Furthermore, the paths from conduct problems to subsequent positive social preference as well as negative social preference were non-significant, although in the latter link again a trend was observed (i.e., *p* ≤ .07.)

**Table 2:** Gene–environment interactions between DRD4 and positive as well as a negative peer social preference in predicting conduct problems and prosocial behavior: fit statistics and nested model comparisons

Model	*χ* ^2^	*df*	CFI	SRMR	Comp.	Δ*χ* ^2^	Δ*df*	*p*
*Positive social preference*								
Total sample								
1. Base model	91.14	33	.95	.05				
2. Time constraints	111.67	47	.94	.05	1 vs. 2	20.37	14	0.119
DRD4-7r versus DRD4-no7								
3. No constraints	172.69	94	.94	.07				
4. Non-hypothesized paths equal	183.71	103	.94	.08	3 vs. 4	9.87	9	0.361
5. GxE: positive social preference → prosocial behavior equal	183.31	104	.94	.08	4 vs. 5	0.06	1	0.805
6. GxE: positive social preference → conduct problems equal	186.87	105	.94	.08	5 vs. 6	5.70	1	0.017
*Negative social preference*								
Total sample								
1. Base model	84.66	33	.95	.06				
2. Time constraints	96.27	47	.95	.05	1 vs. 2	14.84	14	0.389
DRD4-7r versus DRD4-no7								
3. No constraints	184.49	94	.93	.07				
4. Non-hypothesized paths equal	194.34	103	.93	.07	3 vs. 4	9.27	9	0.413
5. GxE: negative social preference → prosocial behavior equal	196.67	104	.92	.07	4 vs. 5	2.85	1	0.091
6. GxE: negative social preference → conduct problems equal	204.01	105	.92	.07	5 vs. 6	9.81	1	0.002

Δ*χ* ^2^ statistics are based on the Satorra–Bentler Chi square difference test

**Table 3:** Coefficients for paths between positive social preference, negative social preference and behavioral phenotypes

Pathways	Estimates			
	*B*	*SE*	*β*	*p*
*Positive social preference*				
Positive social preference predicting prosocial behavior	.47	.12	.14	.000
Prosocial behavior predicting positive social preference	.03	.01	.11	.006
Positive social preference predicting conduct problems	−.17	.10	−.07	.075
Conduct problems predicting positive social preference	.01	.01	.03	.453
*Negative social preference*				
Negative social preference predicting prosocial behavior	−.58	.17	−.10	.001
Prosocial behavior predicting negative social preference	−.01	.01	−.03	.423
Negative social preference predicting conduct problems	.34	.18	.07	.061
Conduct problems predicting negative social preference	.02	.01	.09	.061

As recurring paths were constrained to be similar over time, these results apply to all recurring paths in the model

For prosocial behavioral development, higher positive social preference scores were related to higher subsequent prosocial behavior and more negative social preference scores were related to lower levels of subsequent prosocial behavior. Furthermore, higher levels of prosocial behavior were related to higher levels of subsequent positive social preference, while the paths between prosocial behavior and negative social preference scores were non-significant. These effects were found above and beyond stability paths and cross-sectional correlations, and all estimates were controlled for sex, SES and intervention status. Furthermore, neither the direct effects of DRD4 on social preference, nor the direct relationships between DRD4 and behavioral outcomes were significant.

### Hypothesis 2: Differential Susceptibility of DRD4 to the Environment “For Better and for Worse”

We then tested whether the magnitude of the prospective links between positive and negative social preference scores, prosocial behavior and conduct problems (see Fig. 1 for an illustration), were different for DRD4-7r and DRD4-no7 children (hypothesis 2). Multiple group models were used (DRD4-no7 versus DRD4-7r). Table 2 shows fit indices for models in which all paths were estimated freely between DRD4 groups (models 3), models in which the paths that were not part of our hypothesis were constrained to be equal between the DRD4 groups (models 4), and models in which developmental pathways from social preference to the behavioral outcomes were constrained to be equal between DRD4 groups (models 5 and 6).

Comparisons of fit indices showed evidence for moderation by DRD4 in the link between social preference and subsequent conduct problems only. As can be seen in Table 2, multiple group models in which paths between social preference and subsequent conduct problems were estimated freely between the DRD4-7r and DRD4-no7 groups (models 5), are the best fitting models for children with positive as well as for children with negative social preference scores. Results from analyses for prosocial behavior indicate that neither positive nor negative social preference scores had a differential effect on prosocial behavior as a function of DRD4 (see Table 2).

**Fig. 2:**
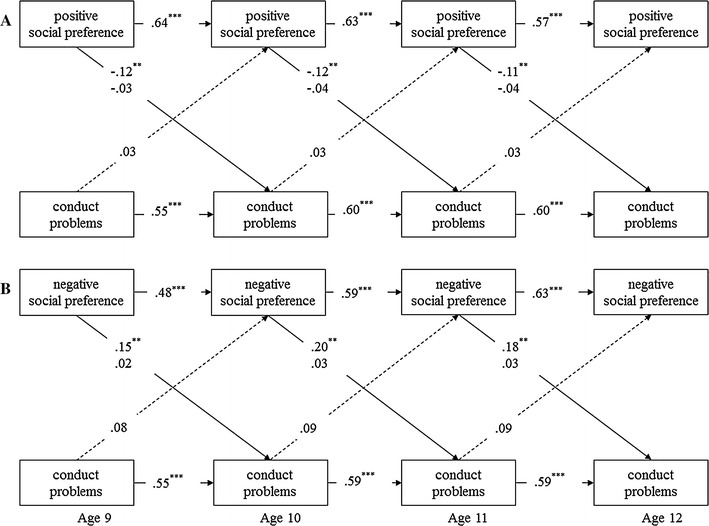
Multiple-group (DRD4-7r vs. DRD4-no7) model of positive social preference (**a**) and negative social preference (**b**) predicting conduct problems. Results are a graphical presentation of models 5. Entries reflect standardized regression coefficients. Paths that were different for the DRD4-7r and DRD4-no7 children have two coefficients: upper entries are estimates for DRD4-7r, lower entries are estimates for DRD4-no7. All entries are controlled for sex, SES and intervention status. *Dashed lines* represent non-significant pathways. *Significant at *p* < .05, **significant at *p* < .01, ***significant at *p* < .001

Estimates of gene–environment interaction effects for conduct problem development are in Fig. 2. Figure 2 shows that positive social preferences scores were prospectively associated with lower levels of conduct problems, but only among DRD4-7r carriers. These effects were mirrored for children with negative social preference scores. That is, being more disliked than liked among peers was associated with more conduct problems, but again only among DRD4-7r carriers. No relation was found between the positive or negative social preference scores and conduct problems for DRD4-no7 children. Note that no G × E interaction effect was found for prosocial behavior. Hence, estimates for associations between social preference and prosocial behavior were similar for the DRD4-7r and DRD4-no7 groups (i.e., similar to findings of the total sample) and can be found in Table 3.

We ran a number of additional tests to test the robustness of our findings. First, potential effects of ethnicity were tested. Specifically, we investigated whether results were similar when only native Dutch children remained in the sample (*N* = 342; *n* = 127 for DRD4-7r, *n* = 215 for DRD4-no7). Results of these tests indicated that removing non-Dutch children from the sample did not influence the results for nested model comparisons. Second, we tested whether the moderating role of DRD4 in the prediction of conduct problems from social preference scores were influenced by the children’s sex. To this end, we investigated the effects of three-way interactions (G × E × sex) on conduct problem and prosocial behavior development, which were all non-significant. Thus the moderation of DRD4 in the association between social preference (positive or negative), prosocial behavior, and conduct problems did not differ between boys and girls. Third, we tested whether the moderating role of DRD4 in the prediction of prosocial behavior and conduct problems from social preference scores was influenced by whether or not children had participated in an intervention. To this end, we investigated the effects of three-way interactions (G × E × intervention status) on conduct problem and prosocial behavior development, which were all non-significant. Thus the moderation of DRD4 in the association between social preference (positive or negative), prosocial behavior, and conduct problems was not dependent upon intervention status. Lastly, we performed post Monte Carlo power analyses (10,000 repetitions) using our sample estimates to calculate the False Discovery Rate (FDR) in our study. Power for our parameters of interest was .0.95 and 1.00 for predicting conduct problem development from positive and negative social preference respectively, which equaled a FDR of 0.05 and 0.01 for positive and negative social preference respectively. This indicates that 5 % of evidence for our hypotheses for positive social preference and 1 % of evidence for our hypotheses for negative social preference with regard to conduct problem development, may actually be type 1 errors.

## Discussion

The main aim of the current study was to investigate whether the dopamine receptor D4 gene (DRD4) moderated the association of positive social preference (i.e., children that were more liked than disliked among classmates) and negative social preference (i.e., children that were more disliked than liked among classmates) among peers with subsequent positive and negative behavioral development. This study was one of the first to investigate differential susceptibility of DRD4 to a common peer environmental experience that covers positive as well as negative aspects of the peer environment. Our first hypothesis that social preference would be related to behavioral development in subsequent years for the group in total was only partially supported. That is, throughout ages 9–12 years children with higher positive social preference scores showed a larger increase in prosocial behavior in subsequent years than children with lower positive social preference scores. This effect was mirrored for negative social preference scores: children with more negative social preference scores showed a larger decrease in prosocial behavior in subsequent years compared to children with less negative social preference scores. Contrary to our expectations, we did not find strong evidence for developmental links between social preference (either positive or negative) and conduct problems in subsequent years for the group in total, although a trend was observed for these developmental links. Our second hypothesis that developmental links between social preference and behavioral outcomes would be moderated by DRD4 “for better and for worse” was also partially supported. As we hypothesized, we found that throughout ages 9–12 years children with higher positive social preference scores showed a larger decrease in subsequent conduct problem development relative to children with lower positive preference scores and that children with more negative social preference scores showed an larger increase in subsequent conduct problem development relative to children with less negative social preference scores, but in both cases only when they carried a DRD4-7r allele. When children did not have this allele, their conduct problem development was not influenced by their social preference among peers. In contrast and contrary to our expectations, prosocial behavioral development was influenced by negative as well as positive social preference among peers regardless of the genetic make-up of the children. Taken together, these findings provide evidence in support of the differential susceptibility hypothesis of DRD4 for conduct problem development, but not for the development of prosocial behavior.

Our findings add to existing knowledge on individual differences in the impact of peer environmental aspects, dependent upon children’s genetic make-up. It concurs with previous studies on bully-victimization (Kretschmer et al. 2013) and peer aggression (DiLalla et al. 2009), in that dopamine-related genes are of importance in understanding the impact of peer environmental factors on behavioral development. Specifically, the results we found in children followed from age 9 to 12 years are in line with DiLalla et al. (2009) who focused on gene–environment interplay in kindergarten and found that children with the DRD4-7 repeat allele were particularly susceptible to their peer environment. Interestingly, Kretschmer et al. (2013) found an opposite effect for adolescents aged 13–18 years of age. That is, their results suggested that it are the DRD4-no7 repeat carriers and not the 7-repeat carriers who are particularly susceptible to the negative as well as the positive environment. A possible explanation for these differential effects for younger versus older children may be that adolescence is a developmental period in which major neurological and biological changes occur, which may influence the effect of DRD4 polymorphisms on behavior/outcomes (Kretschmer et al. 2013). Our findings extend these previous studies by showing that the DRD4-7r allele may not only affect how children respond to these rather extreme peer experiences, but also influences how children respond to common peer evaluations that all children encounter on a daily basis over the elementary school years. In addition, together with the studies of DiLalla et al. (2009) and Kretschmer et al. (2013), the present results warrant attention to the specific developmental period that is under investigation as results from gene–environment interactions may change throughout development.

It is important to note that our findings on gene–environment interplay only held for conduct problems and not for prosocial behavior. In line with differential susceptibility theorizing that DRD4 moderation of environmental effects would be “for better and for worse”, we expected this moderation to be domain general in that both the development of conduct problems and the development of prosocial behavior would be affected. However, our results suggest that this moderation is domain specific. Specifically, our results suggest that DRD4 effects likely depend on the specific environment-behavioral phenotype relation that is investigated. In line with this suggestion, DiLalla et al. (2009) found DRD4 to only moderate the effect of peer aggression on children’s aggressive behavior, but DRD4 did not moderate the effect of peer prosocial behavior on children’s prosocial behavior. As such, the present findings and those of DiLalla et al. (2009) both contribute to a rapidly accumulating body of knowledge that will eventually inform us about the extent to which differential susceptibility effects are domain general or domain specific.

The present findings suggest that Belsky’s (1997) differential susceptibility theory may not only apply to rearing practices, but also to the peer environment. When susceptible children’s position within the peer group is threatened by peer rejection or low preference, one way to strengthen their position is through the use of dominance-oriented social strategies, including aggression (Reijntjes et al. 2013). This is likely to increase individuals’ social dominance position which improves their chances for obtaining attractive resources and (in the future) makes them attractive for mating (Pellegrini and Long 2003), thus improving their chances for reproduction. For susceptible children who are socially preferred by their peers, behaving aggressively to strengthen their dominance position in the peer group is not necessary and given dangerous side-effects (like becoming injured from fighting) may even be undesirable, thus explaining the decrease in subsequent conduct problem development for socially-preferred susceptible children.

Children who were less susceptible (i.e., DRD4-no7 carriers) seemed to be unaffected by their peer environment in that their conduct problem development was not influenced by their social standing among peers. Perhaps children with dopamine-related alleles that are not related to decreased postsynaptic inhibition (e.g., children with DRD4-no7 alleles) have better self-regulatory skills. There is indeed some evidence pointing in this direction (Fan et al. 2003; Fossella et al. 2002; Posner and Rothbart 2009). Better self-regulatory skills may facilitate effective socialization and may enable children to inhibit inappropriate responses like conduct disordered behavior and to behave in accordance with social demands from parents, teachers, and peers. In line with Belsky’s (1997) reasoning regarding differential susceptibility to parenting, it makes evolutionary sense that some children are particularly vulnerable to their peer environment and adapt their behavior accordingly, while others are not influenced by their peers. Future research may elaborate on this suggestion by investigating differential susceptibility of children with DRD4-7r alleles to the peer environment in relation to other behavioral strategies that may strengthen their position in the peer group, such as the combined use of both aggressive and cooperative strategies (Hawley 1999) and behaving as a bully (Olthof et al. 2011).

This study is not without limitations. First of all, although we used a normative sample, the selection of schools was not at random. Children included in our study came from families with higher SES status than is generally reported for the Dutch population (Statistics Netherlands 2012). Furthermore, children whose parents did not consent to having their child’s DNA collected had slightly lower positive social preference scores was well as slightly lower levels of prosocial behavior than children that did participate in the DNA collection. Although the reported differences were small, we cannot be certain that the results generalize to the broader Dutch population. Second, we used teacher reports on children’s prosocial behavior and conduct problems. Teachers may not be aware of these behaviors outside the school context and children may hide certain conduct problems, such as stealing, from their teacher. Although previous studies have indicated that teachers are valid informants of children’s conduct problems and prosocial behavior (Becker et al. 2004; Hart et al. 1994), our results should only be interpreted within the school context. Third, influences of peers as assessed in this study were limited to peers within the classroom. However, poor relations with peers outside the classroom may also affect children’s behavior. Although others have shown that influences of peers outside of the school context are limited for elementary school children (Kupersmidt et al. 1995), we cannot be certain that peers outside the classroom have not influenced our results. Fourth, by investigating the influence of social preference on subsequent behavioral phenotypes while taking into account the stability of these constructs as well as concurrent links between environment and behavior, we were able to identify the actual change in behavioral phenotypes that can be ascribed to peer environment, genetic effects, and their interplay. However, we want to stress that no causality can be inferred from this design. Fifth, although we took both the for better and the for worse side of the differential susceptibility hypothesis into account, we could not directly examine whether the same children who do worse than comparisons in adverse peer environments, also do better when they experience supportive peer environments. Future studies may want to include designs that allow studying the same children in various environmental conditions, such as an experimental study in which the same children encounter peer exclusion as well as inclusion situations (Rutter et al. 2001). In addition, from our study it cannot be inferred which brain processes and neurocognitive functions that are associated with the DRD4 gene account for our differential susceptibility findings. This is of particular importance given the different results that have been found for kindergarten and elementary school children versus older adolescents. Future studies may want to investigate these brain processes and neurocognitive functioning that are associated with differential susceptibility (Ellis and Boyce 2011), ideally within a developmental framework in which potential differences in brain processes and functioning throughout development can be studied. As a last and perhaps most important limitation we want to note that we were not able to directly replicate our results in an independent sample using the same measures. Therefore, our results should be interpreted with caution until replicated.

## Conclusion

The DRD4 7-repeat allele may render children and young adolescents susceptible to their everyday peer environment for better and for worse with regard to subsequent conduct problem development. We found that, throughout ages 9–12 years, children who experienced a more positive peer environment at a given age showed less conduct problem development 1 year later when compared to children who experienced a less positive environment; vice versa, children who experienced a more negative peer environment showed more conduct problem development in subsequent years relative to children who experienced a less negative environment. However, in both situations these effects only held when children had a DRD4-7 repeat allele. Integral strengths of this study were the use of a peer environmental factor that included both a protective and a risk end to assess how a positive and negative daily peer environment may influence the development of conduct problems and prosocial behavior and whether allelic variations within the DRD4 gene may moderate these developmental relations. Other strengths include the use of multiple informants and our longitudinal design. Our findings enhance further understanding of the developmental relationship between youths’ social standing among peers and subsequent behavioral development and advance current knowledge on why some, but not all, children and adolescents are influenced by peer experiences. We suggest that part of the individual differences in responding to the peer environment may be explained by differences in the genetic make-up of these individuals.

Furthermore, our findings have implications for preventive interventions for those children at risk for conduct problem development. The peer environment, regardless whether this environment is positive or negative, affects conduct problem development for those children who are susceptible to it. Preventive interventions that succeed in prohibiting the development of poor peer preference or that improve disliked children’s appraisal among peers to a more neutral level, may decrease the development of conduct problems in susceptible children. Although research on endophenotypes related to susceptibility is still in its infancy, future discoveries of endophenotypes associated with susceptibility may advance the early screening of at-risk children that likely will profit from improvements in peer appraisal. At the same time, as others have suggested (Bakermans-Kranenburg and van IJzendoorn 2011), early detection of those children who likely will not benefit from preventions targeting the peer environment may ideally lead to more individual-based interventions and thus more effective strategies of targeting conduct problem development.

## Appendix 1: Description of Interventions

Approximately 60 % of the children in the present study participated in a preventive intervention targeting problem behavior. Half of the children in the intervention group received the Good Behavior Game intervention (Barrish et al. 1969) and the other 50 % of the intervention children received the PATHS curriculum intervention (Kusché and Greenberg 1994). The interventions were implemented in first and second grade of elementary school.

### Good Behavior Game (GBG)

The GBG (Barrish et al. 1969) is a classroom-based preventive intervention aimed at creating a safe and predictable classroom environment, by promoting adaptive, prosocial classroom behavior. Positively formulated class rules are chosen by the teacher and the students together. To facilitate positive peer interaction, teachers assign children to teams of 4 to 5 members, equally composed of children with and without disruptive behavior. Team members are encouraged to work together and behave adaptively. All teams receive a set of cards at the beginning of the game period in which children work on regular school tasks (e.g., instruction, working alone, reading). Each time a member violates a rule, the teacher takes a card away from that team. Teams as a whole are rewarded (e.g. by extra leisure time, stickers, compliments) for adaptive behavior when at least one card remains at the end of the game period. Game periods lasted between 10 and 60 minutes. During and after the game, compliments are given to the students and teams when deemed appropriate (Dolan et al. 1989).

### PATHS Curriculum

PATHS (Kusché and Greenberg 1994) is a program that targets the development of social and emotional competence in order to decrease the risk of behavioral and social problems. Emotional, cognitive, and social skills are promoted through lessons taught by the teacher. PATHS emphasizes techniques to promote positive interaction amongst students and to reduce peer rejection. For instance, children are taught to adequately express and understand peers’ emotions by using so-called “emotion cards”. Also, children learn problem-solving and anger-management techniques that are generalized throughout the classroom and the school context. Furthermore, the “child of the week” receives particular attention and is allowed to help the teacher throughout the week.

## Appendix 2: Model Specifications and Outcomes Power Analysis Using Monte Carlo Simulations

See Table 4.

**Table 4:** Model specifications and outcomes for a priori multiple-group power analysis using Monte Carlo simulations (*n* repetitions = 10,000)

Path	DRD4-7r *n* = 143			DRD4-no7 *n* = 262		
	Estimate	Coverage	Power	Estimate	Coverage	Power
Autoregressive paths positive social preference	0.60	0.95	1.00	0.60	0.95	1.00
Autoregressive paths prosocial behavior	0.60	0.95	1.00	0.60	0.95	1.00
Autoregressive paths conduct problems	0.60	0.94	1.00	0.60	0.94	1.00
Positive social preference predicting prosocial behavior	0.12	0.94	0.80	0.00	0.95	0.05
Positive social preference predicting conduct problems	−0.12	0.94	0.81	0.00	0.95	0.06
Prosocial behavior predicting positive social preference	0.05	0.95	0.51	0.05	0.95	0.51
Conduct problems predicting positive social preference	−0.05	0.94	0.51	−0.05	0.94	0.51
Correlations positive social preference and prosocial behavior	0.10	0.95	0.22	0.10	0.95	0.36
Correlations positive social preference and conduct problems	−0.10	0.95	0.23	−0.10	0.95	0.38
Correlations prosocial behavior and conduct problems	0.10	0.95	0.23	0.10	0.95	0.37

Estimates of paths reflect standardized regression coefficients. Correlations between constructs reflect residual error correlations. Means of all constructs were estimated to be 0 and variances of all constructs were estimated to be 1. Recurring paths were constrained to be similar over time, hence estimates hold for all recurring paths. Estimates < 0.05 are considered too small to interpret, estimates ≥0.05 are small but meaningful, estimates ≥0.10 are moderate, estimates ≥0.25 are large (Keith 2006)

## Appendix 3: Distribution of the DRD4 Polymorphisms and Assignment to Groups

See Table 5.

**Table 5:** Distribution of the DRD4 polymorphisms and assignment to groups

Genotype	*n*	%
*DRD4-no7 (n* *=* *262)*		
2/2	4	1.0
2/3	3	0.7
2/4	34	8.4
2/5	1	0.2
2/6	1	0.2
3/4	28	6.9
3/5	1	0.2
4/4	173	42.7
4/5	7	1.7
4/6	4	1.0
4/8	5	1.2
5/5	1	0.2
*DRD4-7r (n* *=* *143)*		
2/7	9	2.2
3/7	7	1.7
4/7	111	27.4
5/7	1	0.2
7/7	14	3.5
7/8	1	0.2

DRD4-no7 includes participants with no 7-repeat alleles. DRD4-7r includes participants with at least one 7-repeat allele. The three most common repeat frequencies in our sample were the 4-repeat (66 %), the 7-repeat (19 %), and the 2-repeat (7 %)
